# *ABCC* Gene Variants and Their Effects on Non-Response and Relapse in Pediatric Patients with Central Nervous System Tumors: A Cohort Study

**DOI:** 10.3390/cimb48020205

**Published:** 2026-02-13

**Authors:** Luz María Torres-Espíndola, Juan Carlos Pérez-De Marcos, Manuel de Jesús Castillejos-López, Arnoldo Aquino-Gálvez, Liliana Velasco-Hidalgo, Rocío Cárdenas-Cardós, Armando De Uña-Flores, Marta Zapata-Tarrés, Anjartah Higuera-Iglesias

**Affiliations:** 1 Laboratorio de Farmacología, Instituto Nacional de Pediatría, Ciudad de México 04530, Mexico; 2 Servicio de Oncología, Instituto Nacional de Pediatría, Ciudad de México 04530, Mexico; pdm.jc31018@gmail.com (J.C.P.-D.M.);; 3 Laboratorio de Investigación en Epidemiología y Enfermedades Infecciosas, Instituto Nacional de Enfermedades Respiratorias “Ismael Cosío Villegas”, Tlalpan 4502, Ciudad de México 14080, Mexico; 4 Laboratorio de Biología Molecular, Instituto Nacional de Enfermedades Respiratorias “Ismael Cosío Villegas”, Ciudad de México 14080, Mexico; 5 Servicio de Radiología e Imagen, Instituto Nacional de Pediatría, Ciudad de México 04530, Mexico; 6 Comisión Coordinadora de Institutos Nacionales de Salud y Hospitales de Alta Especialidad, Ciudad de México 14610, Mexico

**Keywords:** *ABC* gene, non-response, relapse, SNV, pediatric patients, central nervous system tumors

## Abstract

The variability in outcomes among individuals is caused by multiple factors, including genetic variations in drug transporter genes known as ABCs. This study investigates the clinical effect of single-nucleotide variants (SNVs) in the *ABCC1*/*MRP1*, *ABCC2*/*MRP2*, and *ABCC4*/*MRP4* genes on the clinical response and relapse of pediatric patients with central nervous system tumors. In a cohort-based association study involving 111 cancer patients, genotyping of *ABCC1*/*MRP1*, *ABCC2*/*MRP2*, and *ABCC4*/*MRP4* was conducted using real-time PCR with TaqMan probes. Treatment response was evaluated using the Response Assessment in Neuro-Oncology (RANO) criteria. Univariate and multivariate analyses were conducted using the Cox proportional hazards (adjusted) model. Multivariate analysis adjusted for sex and age showed a significant association between *ABCC1* r.5540out G>C; rs12921623 in the gene and non-response to treatment in the codominant model [HR] 2.095, 95% CI 1.202–3.650, *p* = 0.009, and in the dominant model [HR] 2.025, 95% CI 1.199–3.421, *p* = 0.008, and an increased risk of relapse in the codominant model [HR] 9.09, 95% CI 1.04–78.85, *p* = 0.04, and in the dominant model [HR] 3.912, 95% CI 1.139–13.436, *p* = 0.03. Furthermore, a significant association was found between *ABCC2* c. 3972 C>T; rs3740066 and relapse in the recessive model [HR] 3.5, 95% CI 1.02–12.17, *p* = 0.04. Our findings indicate that *ABCC1* r.5540 G>C SNV and *ABCC2* c. 3972 C>T SNV are significant predictors of non-response and relapse in this group of pediatric patients with central nervous system tumors.

## 1. Introduction

Cancer treatment-related mortality in children with cancer in low- and middle-income countries can be as high as 45% [1]. Pediatric central nervous system (CNS) tumors are the most common type of solid tumors in children. They are among the leading causes of cancer-related deaths in this age group, accounting for approximately 15–20% of all childhood cancers. The morbidity associated with these tumors can be high and often varies based on their location and histological type [2]. These tumors include a diverse range of biological entities, such as gliomas, medulloblastomas, and ependymomas. Each type displays different clinical behaviors and prognoses, necessitating a multimodal therapeutic approach. This approach involves a combination of surgery, radiation therapy, chemotherapy, targeted therapies, and supportive care aimed at minimizing treatment side effects while preserving neurological function and enhancing long-term quality of life [3]. The integration of safer surgical techniques, targeted radiotherapy, and molecularly targeted treatment strategies has led to improved long-term outcomes [4].

The use of a wide range of drugs has led to cancer cells developing resistance, a phenomenon known as multidrug resistance (MDR), which is currently the leading cause of therapeutic failure in 90% of patients [5,6]. One of the most studied mechanisms of resistance involves ABC transporters. Overexpression of ABC transporters is associated with MDR, leading to adverse clinical outcome, relapse, and mortality [7,8].

Adenosine triphosphate-binding cassette (ABC) subfamily B member 1 (*ABCB1*/*MDR1*), adenosine triphosphate-binding cassette subfamily G member 2 (*ABCG2*/*BCRP*), the adenosine triphosphate-binding cassette subfamily C member 1 (*ABCC1*/*MRP1*) transporters, adenosine triphosphate-binding cassette subfamily C member 2 (*ABCC2*/*MRP2*) and adenosine triphosphate-binding cassette subfamily C member 4 (*ABCC4*/*MRP4*) are transporters that can efflux a wide range of anticancer drugs and cause drug resistance when overexpressed in tumor cells. They are found in the apical membranes of intestinal epithelial cells, hepatocytes, and renal tubular cells. These efflux transporters play a key role in eliminating chemotherapeutic agents, toxic substances, and organic anions from the body, thus contributing to detoxification [9,10]. Drug export can lead to reduced drug concentrations, causing treatment failure, as seen in epilepsy [11,12], breast cancer [13], pancreatic cancer [14], and lung cancer [15].

Single-nucleotide variants (SNVs) have been identified in *ABCC* genes, which may affect the cellular disposition of chemotherapeutic drugs [16]. This can lead to increased or decreased drug efflux and predispose individuals to side effects such as toxicity [17,18,19], ultimately impacting clinical response [20,21].

Multiple drugs currently used in chemotherapy are ABCC substrates. Therefore, it is expected that some SNVs may affect the clinical response. This study investigates the clinical effect of SNVs in the *ABCC1*, *ABCC2*, and *ABCC4* genes on the clinical response and relapse of pediatric patients with central nervous system tumors.

## 2. Materials and Methods

### 2.1. Study Population

The study involved 111 patients under 18 years old with histologically confirmed central nervous system tumors. All cases were referred to the Oncology Department of the National Institute of Pediatrics in Mexico City from November 2018 to November 2020. All patients of this cohort-based association study were treated with neoadjuvant chemotherapy by the protocols used to treat these tumors according to the treatment scheme indicated based on the Mexican guidelines of the Children’s Oncology Group [22]. The treatments administered included ifosfamide, carboplatin, etoposide, vincristine, cyclophosphamide, doxorubicin, cisplatin, and temozolomide. The treatment regimens were categorized as follows: 1. ICE (Ifosfamide, Carboplatin, Etoposide), 2. non-ICE (Vincristine, Carboplatin, Cisplatin, Cyclophosphamide, Etoposide, Temozolomide, Actinomycin D). This research was approved by institutional committees, including ethics, research, and biosafety committees, and is registered under number 061/2018. All patients and/or their parents or guardians provided written informed consent to participate.

### 2.2. DNA Extraction

We collected 5 mL of peripheral blood from each patient with central nervous system tumors. The blood was centrifuged to obtain the leukocyte button, which were lysed. The DNA was extracted using the QIAamp DNA Blood Mini Kit according to the manufacturer’s instructions (Qiagen, Hilden, Germany). We quantified DNA samples and assessed their purity using a BioTek Epoch Microplate Spectrophotometer (Agilent Technologies, Santa Clara, CA, USA), ensuring adequacy for genotyping analyses, and the genomic DNA size was verified by 1% agarose gel electrophoresis.

### 2.3. SNV Genotyping

All single-nucleotide variants (SNVs) were examined by genotyping through allelic discrimination using TaqMan probes. The reaction mixture consisted of 10 ng of genomic DNA, 10 pmol of each primer, 2 pmol of each probe, and 5 µL of 2× master mix (provided by Applied Biosystems, Foster City, CA, USA) in a final volume of 10 µL. The thermocycling process involved 40 cycles: 30 s at 95 °C and 60 s at 60 °C. The PCR plates were read using the Applied Biosystems StepOne instrument. Version 2.2 of the SDS software (provided by Applied Biosystems) was used for genotype discrimination.

All selected SNPs from the NCBI Database of Short Genetic Variations (dbSNP) exhibited minor allele frequencies (MAFs) above 0.02.

The SNVs that were studied include *ABCC1*/*MRP1* rs12921623 (r.5540 G>C), rs12921748 (r.5522 G>A), rs35605 (c.1684 T>C, p.Leu562Leu), *ABCC2*/*MRP2* rs2756109 (r.1658 G>T), rs3740066 (c.3972 C>T, p.Ile1324Ile, *ABCC4*/*MRP4* rs1059751 (c.*4976), rs4148551 (c.*311), and rs3742106 (c.*38).

### 2.4. Outcome: Evaluation of Response to Treatment and Relapse

The evaluation of the response to treatment was carried out by a radiologist according to Response Assessment in Neuro-Oncology (RANO) criteria [23], which consider complete response (CR) as the complete disappearance of all measurable and non-measurable lesions sustained for at least four weeks, without new lesions; partial response (PR) as a decrease ≥50% in the sum of the perpendicular diameters of the measurable lesions compared to the baseline study for at least four weeks; stable disease (SD) when the criteria for complete response, partial response or progression disease (PD) are not met; disease progression with an increase ≥25% in the sum of perpendicular diameters compared to the minimum measurement obtained or concerning the baseline study. Only patients with CR were classified as responders, and patients with PR, SD, and PD were classified as non-responders.

Relapse was defined as the presence of an increase in tumor volume after remission of the disease.

### 2.5. Statistical Analysis

#### 2.5.1. Variable Definition

According to the data distribution, data for qualitative variables were expressed as numbers and percentages, and data for quantitative variables were expressed as median and interquartile range or mean ± standard deviation (SD).

Only patients with complete response were classified as responders, and patients with partial response, stable disease, or tumor progression were classified as non-responders.

SNVs were analyzed using codominant, dominant, and recessive genetic inheritance models: codominant (heterozygous vs. major allele homozygous)/(minor allele homozygous vs. major allele homozygous), dominant (minor allele homozygous + heterozygous vs. major allele homozygous), and recessive (minor allele homozygous vs. heterozygous + major allele homozygous). Each genotype was analyzed independently to determine its association with response to chemotherapy and relapse.

#### 2.5.2. Comparison of Proportions with χ^2^ Test

The χ^2^ test was used to compare the proportions of patients according to the risk and genotypic factors. The time free of non-response to treatment was calculated from the date of diagnosis (biopsy) to the event.

#### 2.5.3. Survival Curves for Each Genotype Under the Heritability Models

The Kaplan–Meier method estimated survival curves for each genotype analyzed under the heritability models. The log-rank test was used to compare time to (non-response to treatment or relapse) between groups.

#### 2.5.4. Multivariate Cox Regression Analysis

These analyses were used to assess whether genotypes participated as prognostic factors for relapse and non-response to treatment adjusting for age, sex, clinical, histopathological characteristics and ICE treatment scheme (ICE: Ifosfamide + Carboplatin + Etoposide). The statistical package used for all analyses was SPSS 21.0 (Statistical Package for Social Sciences, SPSS Inc., Chicago, IL, USA). The *p* values were obtained with the log-rank test after employing the Bonferroni test for multiple testing, and statistical significance was set at *p* < 0.05.

## 3. Results

### 3.1. Clinical Epidemiology of the Central Nervous System Tumor Cohort

A cohort of 111 cases of central nervous system tumors that met the criteria was produced in a tertiary-level hospital. The epidemiological characteristics and tumor groups of the patients are shown in Table 1. The median age was 12 years (_Q25_6–_Q75_ 15 years), and the men’s sex was predominant, N = 61 (55%). The most frequent tumor was medulloblastoma N = 31 (27.93%), high-grade tumors were the most frequent N = 71 (64%), and the non-responder group N = 83 (74.8%) was the most frequent. According to the frequency of treatment schemes, the ICE scheme (ifosfamide, carboplatin, and etoposide) was the most frequent of all the schemes administered, with N = 48 (43.2%).

### 3.2. Survival Analysis

**Figure 1:**
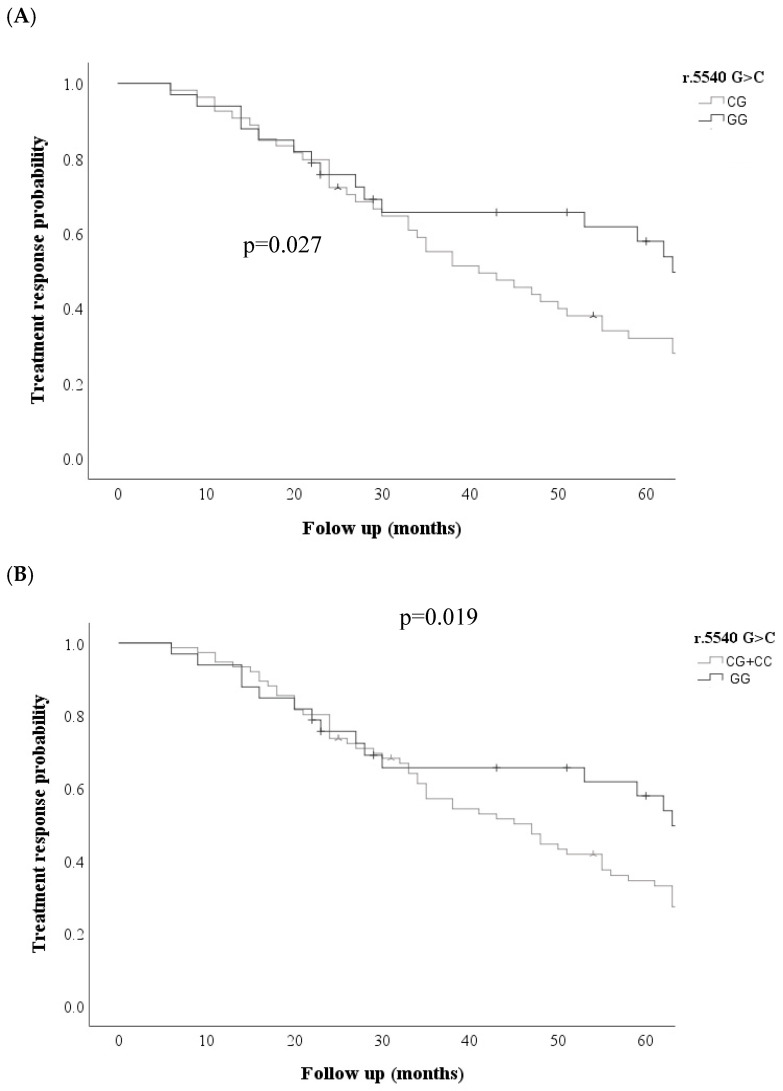
Comparison of treatment response probability curves for the *ABCC1* r.5540 G>C rs12921623 variant between patients with and without SNV. (**A**) Codominant model and (**B**) dominant model.

**Figure 2:**
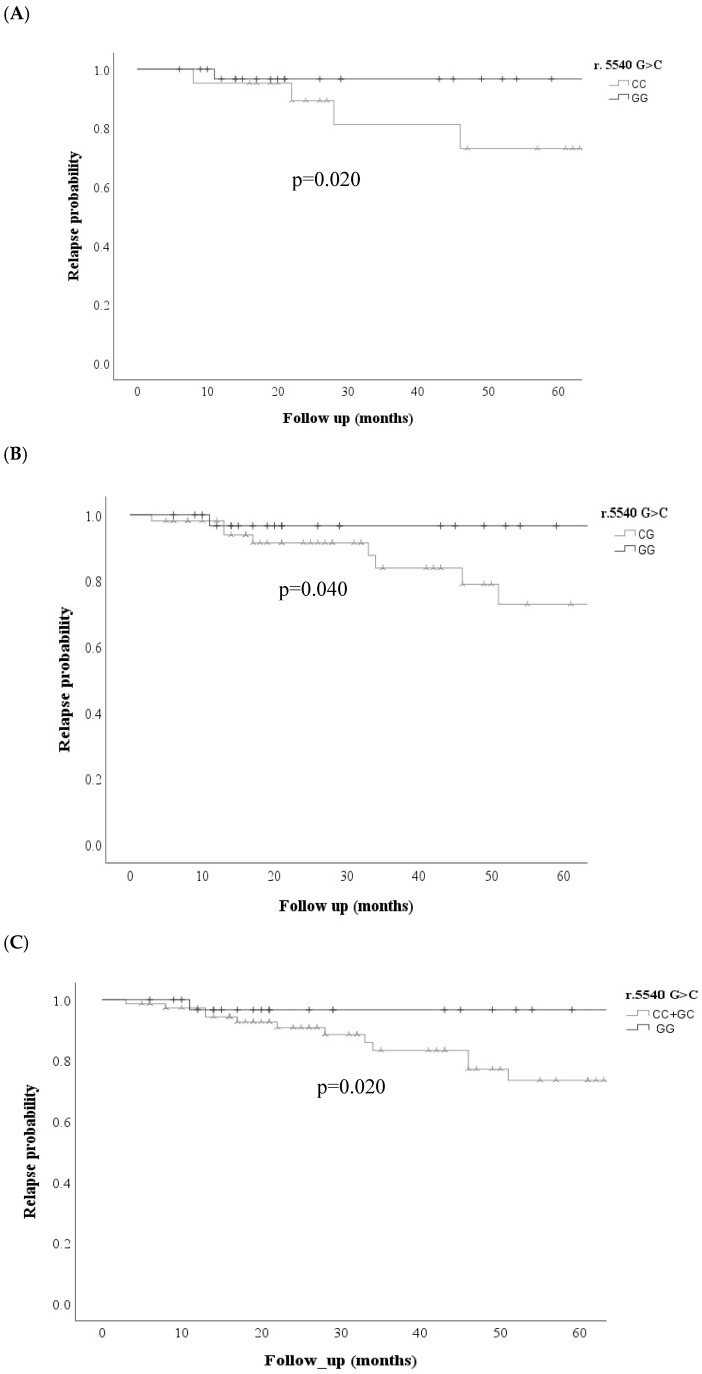
Comparison of relapse probability curves for the *ABCC1* r.5540 C>G rs12921623 variant between patients with and without SNV (**A**) and (**B**) codominant model, (**C**) dominant model.

Kaplan–Meier curves were performed for the *ABCC* transporters in the three models (dominant, codominant, and recessive) for each of the variables analyzed; Kaplan–Meier curves showed that the *ABCC1* r.5540 G>C; rs12921623 gene was associated with an increased probability of non-response to treatment; under the codominant GG + CG model, the *ABCC1* r.5540 G>C; rs12921623 variant was associated with an increased probability of non-response to treatment. (*p* = 0.027) and dominant CC + CG vs. GG (*p* = 0.020) (Figure 1) was also associated with an increased probability of relapse risk in the codominant model: GG vs. CC (*p* = 0.020) GG vs. CG (*p* = 0.04), dominant CC + CG vs. GG (*p* = 0.02) (Figure 2).

**Figure 3:**
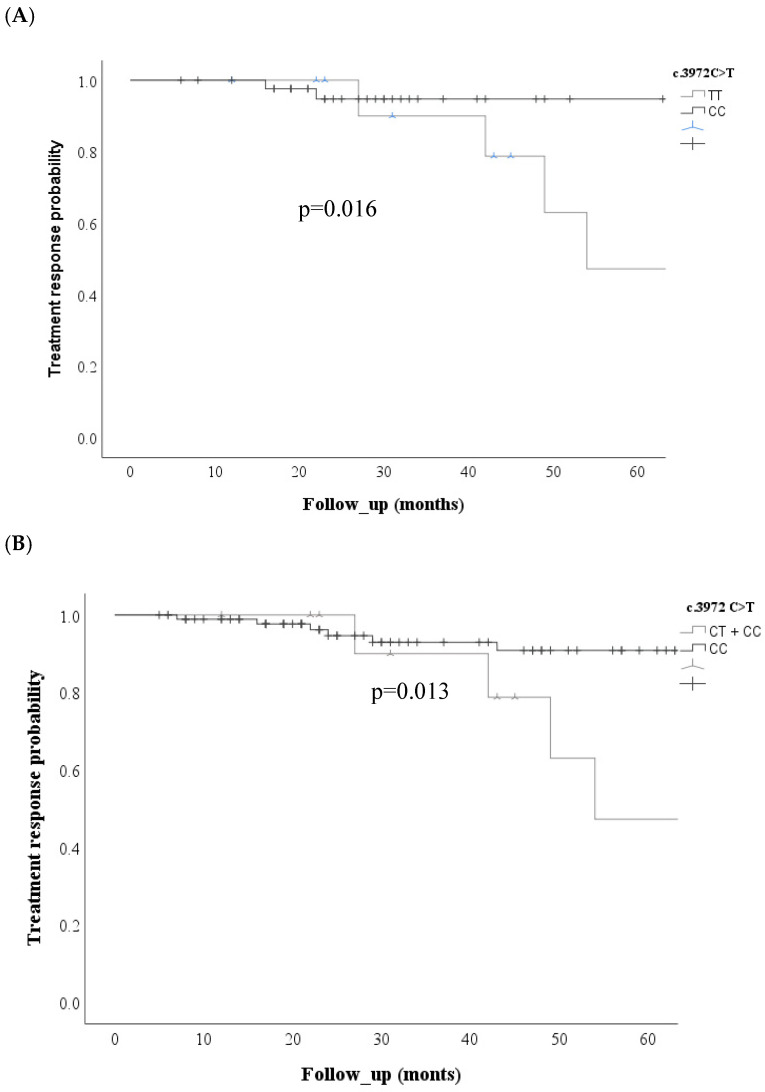
Comparison of treatment response probability curves for the *ABCC2* c. 3972 C>T; rs3740066 variant between patients with and without SNV. (**A**) Codominant model and (**B**) recessive model.

Regarding the *ABCC2* c. 3972 C>T SNV, Kaplan–Meier curves showed an increased risk of non-response to treatment in the codominant model (*p* = 0.016) and the recessive model (*p* = 0.013) (Figure 3).

### 3.3. Correlation Between SNVs ABCC1 r.5540 G>C, ABCC2 c. 3972 C>T and Clinical Response and Relapse

A multivariate analysis was performed to estimate the influence of SNVs adjusted for sex, age, and clinical response. These results are shown in Table 2. A significant association was found between the *ABCC1* r.5540 G>C; rs12921623 gene and non-response to treatment in patients in the codominant model, GG vs. GC [HR] 2.095, 95% CICI95%: 1.202–3.650 *p* = 0.009, and in the dominant model, CC + GC vs. GG [HR] 2.02, 95% CI: 1.199–3.421 *p* = 0.008. No statistically significant association was found for the other variants in the *ABCC2* and *ABCC4* genes.

The influence of these SNVs on relapse was also analyzed, adjusted for sex and age. These results are shown in Table 3. A significant association was found between *ABCC1* r.5540 G>C; rs12921623 variant and relapse in the codominant model: GG vs. CC [HR] 9.09, 95% CI: 1.04–78.85 *p* = 0.04. A significant association was also found in the dominant model: CC + GC vs. GG [HR] 3.912, 95% CI: 1.139–13.436 *p* = 0.03.

A significant association was found between *ABCC2* c. 3972 C>T; rs3740066 variant and relapse in the recessive model: CC vs. CT + CC [HR] 3.5, 95% CI: 1.02–12.17 *p* = 0.04 (Table 3).

## 4. Discussion

To our knowledge, this is one of the few studies describing the clinical significance of SNVs in *ABCC* with clinical non-response and relapse in pediatric patients with central nervous system tumors.

In our study, we found that the *ABCC1* variant r.5540C>G (rs12921623) was significantly linked to treatment non-response and relapse in a cohort of pediatric patients with solid tumors. The multidrug resistance protein ABCC1 functions as an efflux pump located in the plasma membrane, responsible for expelling various endogenous and exogenous substances, including chemotherapeutic agents which may be important in the development of drug resistance in solid tumors.

Although this SNV has not previously been associated with response or relapse, some studies have reported that SNVs in *ABCC1*, in non-coding regions such as introns, alter transporter expression, leading to an altered response to treatment [24].

Warren et al. [25] discovered that two intronic region variants were significantly linked to methotrexate response in psoriasis patients. Carriers of these polymorphisms showed a poorer response to methotrexate treatment.

Tissue expression patterns and wide genetic variability make *ABCC1*/*MRP1* an optimal candidate for use as a marker or member of a multiple-marker panel to predict chemotherapy resistance [26].

There is limited information on this SNV *ABCC2* c. 3972 C>T; rs3740066 in solid tumors in children; most of the existing data focuses on other pathologies discussed below.

The findings of this study also revealed an association of SNV *ABCC2* c. 3972 C>T; rs3740066 with relapse in the recessive model (*p* = 0.04). According to a study in patients with epilepsy, the CT + TT genotypes were associated with increased resistance to antiepileptic drugs in people with epilepsy compared to the CC genotype (*p* = 0.038) [27]. According to a study in patients with epilepsy, the CT + TT genotypes were associated with increased resistance to antiepileptic drugs in people with epilepsy compared to the CC genotype [28]. This variant has been associated with reduced promoter activity and lower *ABCC2* mRNA levels [29,30].

On the other hand, in colorectal neoplasms, the TT genotype was associated with greater severity of neurotoxicity syndromes when treated with fluorouracil, leucovorin, and oxaliplatin in individuals with colorectal neoplasms compared to CC + CT genotypes [31].

Hegyi et al. [32]. found that the TT genotype was associated with reduced methotrexate concentrations in children with osteosarcoma compared to the CC + CT genotypes 

In patients with sarcomas treated with anthracyclines, the TT genotype was associated with shorter overall survival than CC + CT genotypes [33]. Thishya K. et al. (2021) found that the variant was associated with mortality in renal transplant patients [34]. Numerous studies suggest that ABCC expression may be an indicator of chemotherapy efficacy [35,36,37,38,39].

Two major families of transporters are present in the blood–brain barrier: solute transporters (SLCs) and ATP-binding cassette (ABC) transporters. Efflux transporters play a crucial protective role by removing metabolic waste and preventing the entry of potential toxins and most therapeutic drugs that could affect the brain. The expression of transporters such as *ABCB1*, *ABCC2*, *ABCG2*, and *ABCC4* at the blood–brain barrier is well established [40,41,42].

Resistance to chemotherapy can develop through several mechanisms. One suggested mechanism involves single-nucleotide variants (SNVs) that affect the overexpression of ABC transporters. This overexpression can lead to increased drug efflux, which lowers the concentration of the drug in the cytoplasm. As a result, the drug’s effectiveness is reduced, potentially leading to the development of a drug-resistant phenotype. This variability may help explain the differences in patient responses and levels of toxicity [2,43,44,45].

Low expression levels of these MDR transporters, which may be caused by abnormalities in mRNA or defects in drug export proteins, can lead to the accumulation of drugs within cells. This occurs due to reduced export and slower removal of chemotherapeutic agents, potentially resulting in a prolonged cytotoxic effect. These conditions can negatively impact treatment response and decrease patient survival.

From a clinical perspective, our findings support the growing interest in pharmacogenetic approaches in pediatric oncology. Genetic variability in *ABC* transporters may not only influence treatment response and relapse risk but could also contribute to interindividual differences in drug exposure and toxicity. Pediatric pharmacogenomic research indicates that variability in drug transporter expression and function can partly explain differences in chemotherapy-related toxicities and responses, highlighting the potential value of incorporating transporter genotyping into individualized treatment strategies [46]. Furthermore, systematic reviews in pediatric solid tumors have shown associations between transporter gene variants (including *ABCC2* and related *ABC* genes) and specific toxicity outcomes, underscoring the need to consider genetic factors when optimizing drug selection and dosing in children [47]. In pediatric patients, where developmental pharmacokinetics and narrow therapeutic windows are critical considerations, genetic testing of drug transporters may represent an additional tool to support individualized treatment decisions. Although our study was not designed to evaluate dose adjustments or toxicity outcomes, the observed associations suggest that *ABCC* genotyping could complement clinical and therapeutic factors in future studies aimed at optimizing drug selection and dosing strategies while minimizing adverse effects.

Finally, we acknowledge some limitations of our study, including cohort heterogeneity and the variety of drugs used in treatment. This underscores the need for standardized guidelines for managing these tumors. Therefore, our findings should be viewed as associative and designed to generate hypotheses, rather than implying a causal relationship.

## 5. Conclusions

Our data suggests that genetic variants *ABCC1* (rs12921623) and *ABCC2* (rs3740066) may be crucial in predicting non-response and relapse in pediatric patients with central nervous system tumors.

The study of SNV in drug transport genes has the potential to provide valuable information on predicting therapeutic responses to the use of different xenobiotics in the Mexican population and relapse. When genotyping response biomarkers for making more accurate predictions regarding reactions to chemotherapy drugs, it is important to consider that tumor heterogeneity and the different mechanisms of drug action can alter the results. Therefore, further studies are needed to confirm our findings.

## Figures and Tables

**Table 1:** Characteristics of the study cohort.

Characteristic	N (%)
Sex	
Men	61 (55)
Women	50 (45)
Age at diagnosis (years)	
Median	12
Embryonic	10
Glioma	8
Germinal	14
Histologic lineage	
Glioma	61 (55.0)
High-grade	21 (18.9)
Low-grade	40 (36.1)
Embryonic	39 (35.1)
Medulloblastoma	31 (27.9)
PNET	4 (3.6)
Pineoblastoma	3 (2.7)
Atypical teratoid/rhabdoid	1 (0.9)
Germinal	8 (7.2)
Other	3 (2.7)
Clinical response	
Complete response	22 (19.8)
Partial response	14 (12.6)
Stable disease	24 (21.6)
Progression	51 (45.9)
Treatment scheme	
ICE	48 (43.2)
No ICE	63 (56.8)
Relapse	
Present	21 (18.9)
Absent	90 (81.1)

**Table 2:** Multivariate analysis using the Cox proportional hazards model for non-response.

Gene	Codominant				Dominant		Recessive	
	HR (95% CI)	*p*-Value	HR (95% CI)	*p*-Value	HR (95% CI)	*p*-Value	HR (95% CI)	*p*-Value
*ABCC1*								
r.5540 G>C	1.778 (0.918–3.44)	0.088	2.095 (1.202–3.650)	0.009 *	2.02 (1.199–3.421)	0.008 *	1.22 (0.656–1.919)	0.678
Age	0.962 (0.908–1.019)	0.190	0.928 (0.882–0.977)	0.004	0.931 (0.892–0.972)	0.001	0.928 (0.886–0.971)	0.001
Sex	0.590 (0.297–1.171)	0.132	0.640 (0.392–1.044)	0.074	0.692 (0.433–1.05)	0.082	0.678 (0.440–1.044)	0.078
*ABCC2*								
c. 3972 C>T	0.710 (0.317–1.588)	0.404	0.907 (0.565–1.456)	0.685	0.895 (0.554–1.384)	0.568	0.845 (0.401–1.782)	0.658
Age	1.026 (0.531–1.982)	0.939	0.733 (0.459–1.171)	0.194	0.941 (0.901–0.963)	0.007	0.939 (0.900–0.9809)	0.004
Sex	0.953 (0.900–1.009)	0.097	0.941 (0.898–0.986)	0.011	0.734 (0.472–1.142)	0.170	0.728 (0.468–1.131)	0.158

HR: hazard ratio, CI: confidence interval. * Statistical significance was calculated using a multivariate Cox regression.

**Table 3:** Multivariate analysis using the Cox proportional hazards model for relapse.

Gene	Codominant				Dominant		Recessive	
	HR (95% CI)	*p*-Value	HR (95% CI)	*p*-Value	HR (95% CI)	*p*-Value	HR (95% CI)	*p*-Value
*ABCC1*								
r.5540 G>C	9.09 (1.04–78.85)	0.04 *	8.161 (0.909–73.29)	0.06	3.912 (1.139–13.436)	0.03 *	2.69 (0.89–8.11)	0.07
Age	0.53 (0.09–2.66)	0.42	0.432 (0.114–1.629)	0.21	0.924 (0.847–1.007)	0.07	0.98 (0.88–1.08)	0.70
Sex	1.01 (0.87–1.17)	0.88	0.942 (0.803–1.104)	0.45	1.18 (0.502–2.773)	0.70	0.60 (0.20–1.76)	0.35
*ABCC2*								
c. 3972 C>T	0.37 (0.08–1.56)	0.17	1.26 (0.36–4.42)	0.71	1.06 (0.35–3.19)	0.91	3.5 (1.02–12.17)	0.04 *
Age	0.99 (0.86–1.14)	0.93	0.97 (0.85–1.14)	0.66	0.99 (0.89–1.119	0.93	0.98 (0.87–1.10)	0.76
Sex	0.46 (0.09–2.38)	0.35	2.36 (0.65–8.57)	0.19	1.58 (0.54–4.57)	0.39	1.03 (0.32–3.30)	0.95

HR: hazard ratio, CI: confidence interval. * Statistical significance was calculated using a multivariate Cox regression.

## Data Availability

The raw data supporting the conclusions of this article will be made available by the authors on request.

## Abbreviations

MDR	multidrug resistance
ABC	Adenosine triphosphate-binding cassette
SNV	Single-nucleotide variants
ICE	Ifosfamide, Carboplatin, Etoposide.
MAF	Minor allele frequencies
RANO	Response Assessment in Neuro-Oncology
CR	Complete response
PR	Partial response
SD	Stable disease
PD	Progression disease
SD	Standard deviation
HR	Hazard ratio
CI	Confidence interval
PNET	Primitive neuroectodermal tumor
